# Design of a US28 ORF Deletion Virus in a Temperature-Sensitive Cytomegalovirus Strain Fails to Promote Lytic Replication in Hematopoietic Cells

**DOI:** 10.3390/v14061280

**Published:** 2022-06-12

**Authors:** Benjamin A. Krishna, Amanda B. Wass, Eain A. Murphy, Christine M. O’Connor

**Affiliations:** 1 Genomic Medicine, Infection Biology Program, Global Center for Pathogen & Human Health Research, Lerner Research Institute, Cleveland Clinic, Cleveland, OH 44195, USA; back2@cam.ac.uk (B.A.K.); wassa@ccf.org (A.B.W.); 2 Microbiology and Immunology, Upstate Medical University—State University of New York (SUNY), Syracuse, NY 13210, USA; murphye1@upstate.edu

**Keywords:** cytomegalovirus, CMV, latency

## Abstract

Human cytomegalovirus (CMV) is a ubiquitous pathogen that latently resides in hematopoietic cells. Latently infected individuals with dysfunctional immune systems often experience CMV reactivation, which can cause devastating disease and mortality. While factors dictating the balance between latency and reactivation are not completely understood, CMV US28 is required for maintaining latent infection, and viral mutants that alter US28 function result in a lytic-like, rather than latent, infection in hematopoietic cells. In turn, viral lytic factors alter the host cell, making it challenging to characterize the US28-specific changes in the cellular milieu. To circumvent this, we generated a temperature-sensitive TB40/E recombinant virus, TB40/E*gfp*C510G (*ts*C510G), into which we engineered an amino acid change at position 510 (C510G) of IE2, as previously described in the CMV Towne strain. Using *ts*C510G, we then deleted the US28 ORF, termed *ts*C510G-US28Δ. Consistent with previous findings, *ts*C510G-US28Δ fails to undergo latency in Kasumi-3 cells at the permissive temperature. However, parallel cultures maintained at the non-permissive temperature showed a significant reduction in infectious center frequency, as measured by limiting dilution assay. Thus, we generated a new US28 mutant virus for use as a tool to study US28-specific changes in latently infected hematopoietic cells in the absence of induced lytic replication.

## 1. Introduction

The human betaherpesvirus, cytomegalovirus (CMV), is a ubiquitous pathogen that, once acquired, establishes a lifelong latent infection in cells of the hematopoietic compartment. While CMV infection remains asymptomatic in healthy individuals, immunocompromised and immunosuppressed patients often undergo CMV reactivation, leading to lytic replication, and the dissemination of the virus often leads to disease. Approved anti-viral therapies that target the active phase of infection are effective, but can lead to viral resistance and toxicity (e.g., refs. [1,2,3,4]). Thus, there is a significant at-risk patient population who will suffer from complications of CMV-associated disease following reactivation, which can result in significant morbidity and mortality.

The host and viral factors that regulate the balance between latency and reactivation are still not completely understood. CD34^+^ hematopoietic progenitor cells (HPCs) and circulating monocytes latently harbor CMV, and differentiation to macrophages or dendritic cells, along with chromatin remodeling, triggers reactivation [5,6,7]. Many labs dedicated significant resources to bolstering our understanding of these phases of infection, and it is clear that the latent infection of hematopoietic cells initiates cell signaling pathways, transcription factor regulation, and the differential expression of host and viral factors that support CMV latency [5,6,7,8]. Signaling by the viral G protein-coupled receptor (GPCR), US28, is required to maintain CMV latent infections [9,10,11,12,13,14,15,16], during which it alters host cell signaling axes that ultimately lead to the silencing of the major immediate early (MIE) locus, which is responsible for expression of the viral lytic transcripts, *UL123* and *UL122* [9,11,13,15,17]. US28 is a potent signaling molecule with cell-type-specific properties [17]; thus, our understanding of how US28 alters the host cell milieu in the context of the lytic infection of cells that support lytic infection (e.g., fibroblast, endothelial, epithelial cells) will likely differ from this protein’s function (s) in hematopoietic cells that support latency.

Studies on US28′s role during latency are ongoing, and many groups, including our own, have generated viral recombinants to study its function [10,11,12,13,15,18]. We and others have used bacterial recombineering to mutate US28 in the context of bacterial artificial chromosome (BAC)-derived virus strains, generating US28 complete open reading frame (ORF) deletion mutants, stop mutants, G protein-coupling mutants (R129A), and ligand-binding mutants (Y16F, ΔN). Indeed, the use of these mutant viruses revealed US28’s necessity during viral latency, as any aberration in wild type US28 expression leads to an infection that favors lytic replication [9,10,11,12,13,14,15,16]. However, there remains a caveat to further understanding the cellular factors US28 manipulates during the latent infection of hematopoietic cells, such as CD34^+^ HPCs; the infection of these cells with a US28 mutant virus results in a lytic-like phenotype, in which the host cell is altered by viral lytic factors. This makes it difficult to characterize cellular alterations specific to the biological functions of US28 in the background of active lytic transcription. Thus, to understand US28-dependent changes, it is important to separate what happens in a hematopoietic cell infected with a virus lacking US28 versus what happens in a cell due simply to lytic infection.

The MIE promoter (MIEP) drives the transcription of *UL123*, which encodes the immediate early protein, IE1 (also known as IE72), and *UL122*, which encodes IE2 (also known as IE86), an essential protein for viral lytic replication [19]. IE2 transactivates early and late gene promoters [19,20,21], thus acting as a gatekeeper to successful lytic infection. Previous work from Heider et al. demonstrated that mutation of the cysteine to glycine at position 510 (C510G) in IE2 rendered this protein temperature-sensitive, which in turn prevented the efficient lytic replication of the lab-adapted Towne strain of CMV at the non-permissive temperature (39.5 °C) [22]. Therefore, we reasoned that the C510G mutation might also allow mutant viruses favoring lytic replication in hematopoietic cells, such as US28 mutant viruses, to instead retain a more “latent-like” phenotype if maintained at the non-permissive temperature. However, testing this would require generating a similar recombinant virus in a clinical CMV strain, as lab-adapted strains, including Towne, fail to infect cells, including epithelial and endothelial cells as well as hematopoietic cells, in which CMV undergoes latency [23]. Thus, herein, we show that the *UL122* C510G mutation also inefficiently renders the BAC-derived clinical strain, TB40/E*gfp* replicate in lytically infected fibroblasts cultured at the non-permissive temperature. We also show that the temperature-sensitive mutant, TB40/E*gfp*-C510G (*ts*C510G), undergoes latency and reactivation in primary CD34^+^ HPCs at the permissive temperature, while reactivation at the non-permissive temperature fails to produce infectious centers. Building on this key resource, we deleted the entire US28 ORF from *ts*C510G, termed TB40/E*gfp*C510G-US28Δ (*ts*C510G-US28Δ), which mimics the parental temperature-sensitive virus in terms of lytic replication at both permissive and non-permissive temperatures. Importantly, while *ts*C510G-US28Δ fails to undergo latency in Kasumi-3 cells infected under latent conditions at the permissive temperature, the parallel cultures of these infected cells maintained at the non-permissive temperature resulted in a significant reduction in the frequency of infectious centers by the limiting dilution assay. In summary, we report the generation of a temperature-sensitive, US28Δ mutant virus in the BAC-derived TB40/E backbone to use as a tool to study US28-specific changes in hematopoietic cells that support latency in the absence of induced lytic replication.

## 2. Materials and Methods

### 2.1. Cells

Primary newborn human fibroblasts (NuFF-1; GlobalStem, Rockville, MD, USA) were cultured in Dulbecco’s modified Eagle medium (DMEM), supplemented with 10% fetal bovine serum (FBS), 2 mM L-glutamine, 0.1 mM non-essential amino acids, 10 mM HEPES, and 100 U/mL each of penicillin and streptomycin. Kasumi-3 cells (ATCC) were cultured in Roswell Park Memorial Institute (RPMI) 1640 medium (ATCC, catalog no. 30-2001), supplemented with 20% FBS, 100 U/mL each of penicillin and streptomycin, and 100 μg/mL gentamicin and maintained at a density of 5 × 10^5^–1 × 10^6^ cells/mL. Primary CD34^+^ hematopoietic progenitor cells (HPCs) were isolated from de-identified cord blood samples (Abraham J. & Phyllis Katz Cord Blood Foundation d.b.a. Cleveland Cord Blood Center & Volunteer Donating Communities in Cleveland and Atlanta) by magnetic separation, as previously described in detail [24]. M2-10B4 (MG3) and S1/S1 murine stromal cells were provided as gifts from Terry Fox Laboratories, BC Cancer Agency, Vancouver, BC, Canada. MG3 cells were propagated in RPMI 1640, supplemented with 10% FBS and 100 U/mL each of penicillin and streptomycin. S1/S1 cells were cultured in Iscove’s modified Dulbecco’s medium (IMDM), supplemented with 10% FBS, 1 mM sodium pyruvate, and 100 U/mL each of penicillin and streptomycin. MG3 and S1/S1 cells were plated at a 1:1 ratio (~1.5 × 10^5^ cells per cell type) onto collagen-coated (1 mg/mL) 6-well plates in human long-term culture media (hLTCM; MyeloCult H5100 (Stem Cell Technologies) supplemented with 1 μM hydrocortisone, and 100 U/mL each of penicillin and streptomycin). The following day, the cells were irradiated using a fixed source ^137^Cesium, Shepherd Mark I Irradiator at 20 Gy, and allowed to recover for 24 h, after which they were used as feeder cells for the CD34^+^ HPC latency assays described in further detail below.

Kasumi-3 and CD34^+^ cell survival was evaluated at 32.5 °C and 39.5 °C. Kasumi-3 cells were cultured as above, and CD34^+^ primary cells were maintained in hLTCM media, without the stromal cell feeder layer. At 7 days post-plating, cell viability was evaluated using trypan blue.

### 2.2. Viruses

Bacterial artificial chromosome (BAC)-derived TB40/E*gfp* was used throughout as the wild type (WT) virus and previously described [25]. Using *IscE* recombineering protocols, described in detail elsewhere [25,26], TB40/E*gfp* was used to generate TB40/Egfp-tsC510G (tsC510G), and the sequence was verified by Sanger sequencing. BAC DNA of tsC510G was then isolated, and the BAC DNA was moved from GS1783 to SW105 bacterial cells, then used as the backbone to generate TB40/Egfp-tsC510G-US28Δ (tsC510G-US28Δ) by galK recombineering, as described previously [27]. All primers used to generate the recombinants and verify the DNA sequences are listed in Table 1.

Viral stocks were essentially generated as described elsewhere (e.g., [25]). Viral stocks were titered by 50% tissue culture infectious dose assay (TCID_50_) on naïve NuFF-1 cells. The temperature-sensitive mutants tsC510G and tsC510G-US28Δ were each grown at the permissive temperature (32.5 °C) to generate the stocks and determine titers.

### 2.3. Multistep Growth Analyses

To evaluate viral lytic growth, NuFF-1 cells were infected at a multiplicity of 0.01 TCID_50_/cell for 1 h at the permissive temperature (32.5 °C), rocking the cultures gently every 15 min. Inocula were then removed, cells were washed three times with phosphate-buffered saline (PBS), and fresh media were added (DMEM, supplemented with 10% newborn calf serum, 100 U/mL each of penicillin and streptomycin). One set of infections was maintained at 32.5 °C (permissive), while the other was cultured at 39.5 °C (non-permissive). Extracellular virus was collected at the times indicated in the text over a 16 d time course. Virus from each infection was then titrated on naïve NuFF-1 cells, and titers were quantified by TCID_50_ assay, during which all cultures were maintained at the permissive temperature (32.5 °C).

### 2.4. Infection of Kasumi-3 and CD34^+^ Cells

Kasumi-3 cells were infected as described before [10,11,25]. Briefly, cells were maintained in X-VIVO15 (Lonza) for 2 d and then infected at a multiplicity of 1.0 TCID_50_/cell by centrifugal enhancement (1000× *g*, 35 min, room temperature) at 5 × 10^5^ cells/mL in X-VIVO15. Infected cells were allowed to recover overnight, and the next day, they were treated with trypsin to remove any virus that had not entered the cell. Cells were next cushioned onto Ficoll-Paque (GE Healthcare Life Sciences) to remove residual virus and debris, washed three times with PBS, and replated in X-VIVO15 at 5 × 10^5^ cells/mL.

Isolation of CD34^+^ HPCs is described in detail elsewhere [24], and these cells were infected at a multiplicity of 2.0 TCID_50_/cell, as previously described [10,11,25,28], in infection media consisting of IMDM supplemented with 10% BIT9500 serum substitute (Stem Cell Technologies), 2 mM L-glutamine, 20 ng/mL low-density lipoproteins, and 50 μM 2-mercaptoethanol. The following day, cultures were washed three times in PBS and replated in 0.4 μm-pore transwells (Corning) over irradiated murine stromal cells in hLTCM, as detailed above.

### 2.5. Extreme Limiting Dilution Assay

Latency and reactivation were assessed using extreme limiting dilution assay (ELDA), essentially as previously described [24]. For CD34^+^ HPCs, cells were infected under latent conditions at the temperatures indicated in the text. At 7 days post-infection (dpi), cells were then serially diluted two-fold onto naïve NuFF-1 fibroblasts in the presence of either hLTCM to maintain latency or reactivation media (RPMI supplemented with 20% FBS, 10 mM HEPES, 1 mM sodium pyruvate, 2 mM _L_-glutamate, 0.1 mM 0.1 mM non-essential amino acids, 100 U/mL each of penicillin and streptomycin with 15 ng/mL each of IL-6, G-CSF, GM-CSF and IL-3, all from R&D Systems). CD34^+^ HPCs were co-cultured with the naïve fibroblasts for 14 d at the temperatures indicated in the text. Infected Kasumi-3 cells were maintained in media favoring latency (X-VIVO15) for 7 d at the temperatures indicated in the text. Infected cells were serially diluted two-fold onto naïve NuFF-1 cells as above and cultured for 14 d at the temperatures indicated in the text. For both CD34^+^ HPCs and Kasumi-3 cells, the frequency of infectious centers was quantified using ELDA software [28].

## 3. Results

### 3.1. tsC510G Displays Impaired Lytic Growth at the Non-Permissive Temperature

**Figure 1:**
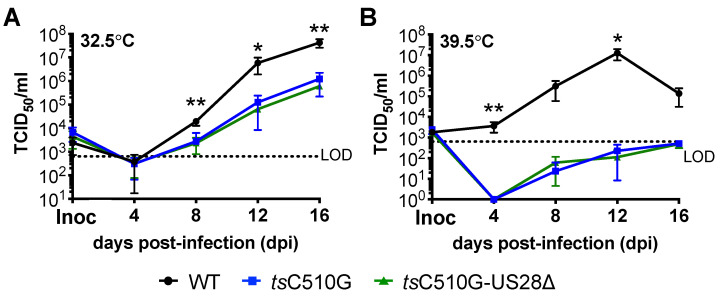
*ts*C510G-US28∆ replicates to similar titers as *ts*C510G in lytically infected fibroblasts. NuFF-1 cells were infected (moi = 0.01 TCID_50_/cell) as indicated at the (**A**) permissive (32.5 °C) or (**B**) non-permissive (39.5 °C) temperature. Extracellular virus was collected over a 16 d time course and titered on naïve NuFF-1 cells at the permissive temperature (32.5 °C) by TCID_50_. Error bars indicate standard deviation, and statistical significance was calculated using one-way ANOVA with Tukey’s multiple comparison test: (**A**) * *p <* 0.05 (WT vs. *ts*C510G; WT vs. *ts*C510G-US28∆); ** *p* < 0.01 (WT vs. *ts*C510G; WT vs. *ts*C510G-US28∆); all other comparisons were not significant. (**B**) * *p* = 0.0203 (WT vs. *ts*C510G; WT vs. *ts*C510G-US28∆); ** *p =* 0.0169 (WT vs. *ts*C510G; WT vs. *ts*C510G-US28∆); day 8: *p* = 0.0869 (for each, WT vs. *ts*C510G; WT vs. *ts*C510G-US28∆); day 16: *p* = 0.0765 (WT vs. *ts*C510G) and *p* = 0.0764 (WT vs. *ts*C510G-US28∆). LOD, level of detection. *n* = 3; representative experiment is shown, with 3 technical replicates per time point.

To generate the temperature-sensitive mutant in the BAC-derived TB40/E*gfp* backbone, we leveraged bacterial recombineering, using the I-SceI method [25,26] to mutate the UL122 gene encoding IE2. To this end, we introduced a single amino acid substitution at position 510 from cysteine to glycine (C510G). Heider et al. previously characterized this mutation in the Towne strain of CMV, showing C510G results in a temperature-sensitive IE2, leading to the failure of this protein to activate early promoters, and thus a progression through the lytic life cycle at the non-permissive temperature of 39.5 °C [22]. Similar to this study on the mutant virus, our newly generated temperature-sensitive virus, TB40/EgfpC510G (tsC510G), lytically replicated in fibroblasts maintained at the permissive temperature of 32.5 °C, although not as efficiently as TB40/E*gfp* (WT) (Figure 1A). This is also consistent with findings from Heider et al. [22]. However, in parallel-infected cells cultured at the non-permissive temperature of 39.5 °C, tsC510G displayed a significant growth defect (Figure 1B). We also generated an additional virus, in which we deleted the entire US28 ORF from tsC510G, yielding TB40/EgfpC510G-US28Δ (tsC510G-US28Δ). At the permissive temperature, tsC510G-US28Δ grows to similar titers as its parental virus, C510G, in lytically infected fibroblasts (Figure 1A). This is also consistent with previous findings showing that US28 is dispensable for lytic replication (e.g.*,* ref. [29]). Similar to tsC510G, tsC510G-US28Δ also fails to effectively replicate at the non-permissive temperature (Figure 1B). Thus, similar to previous findings [22], our data show that the IE2 C510G mutation results in a virus that fails to efficiently and lytically replicate at the non-permissive temperature.

### 3.2. tsC510G-Infected Primary CD34^+^ Hematopoietic Progenitor Cells Reactivate from Latency

**Figure 2:**
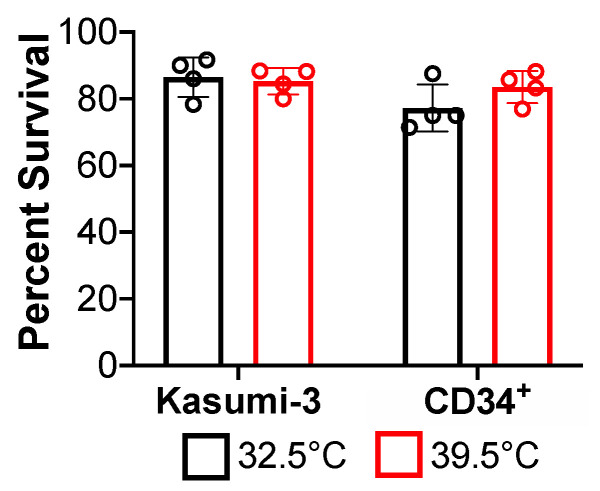
Kasumi-3 and CD34^+^ cells survive in culture at 32.5 °C and 39.5·°C. Each cell type was cultured for 7 d at the indicated temperature, and cell viability was assessed using trypan blue staining. Data are shown as percent survival relative to the original number of cells plated. Error bars indicate SD; representative data shown (*n* = 3 biological replicates, each with *n* = 4 technical replicates).

We hypothesized that since *ts*C510G fails to lytically replicate efficiently at 39.5 °C, reactivation at this non-permissive temperature would also be inefficient following the latent infection with this mutant. We began by ensuring each cell type we used in our assays, Kasumi-3 cells (a CD34^+^ cell line, that supports latency and reactivation [25]) and primary cord blood-derived CD34^+^ HPCs, would survive at both the permissive (32.5 °C) or non-permissive (39.5 °C) temperatures. To this end, we cultured each cell type at either 32.5 °C or 39.5 °C and observed no significant growth or viability defects in either Kasumi-3 cells or CD34+ HPCs (Figure 2). Next, we tested the ability of *ts*C510G to undergo latency and reactivation in primary CD34^+^ HPCs. To this end, we isolated CD34^+^ HPCs from the combined cord blood from two de-identified donors, allowing us to alleviate concerns regarding donor variability.

**Figure 3:**
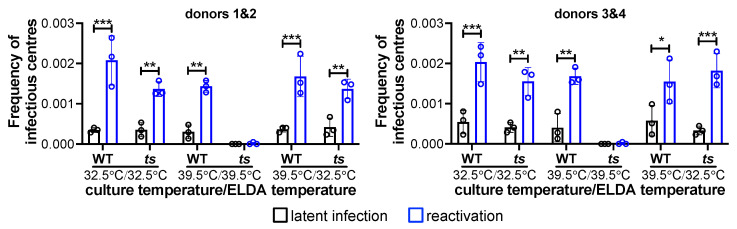
CD34^+^ HPCs infected with *ts*C510G at the non-permissive temperature successfully reactivate from latency when shifted to the permissive temperature. CD34^+^ HPCs were infected (moi = 2.0 TCID_50_/cell) with WT or *ts*C510G (*ts*) and maintained in media favoring latency (latent infection, black bars) at the indicated culture temperature (7 d). Half of each infected population was then cultured in reactivation media (reactivation, blue bars) at the indicated reactivation temperature. Each infected population was co-cultured with naïve NuFF-1 cells (14 d), and the frequency of infectious centers was calculated by ELDA. Two biological replicates are shown, each of which contained pooled donor cells. Each data point (circles) represents a technical replicate (*n* = 3 per condition). Error bars indicate SD; statistical significance was calculated using two-way ANOVA followed by Tukey’s post hoc analysis. * *p* < 0.05, ** *p* < 0.01, *** *p* < 0.001.

We then infected these cells with either WT or *ts*C510G under latent conditions at either 32.5 °C or 39.5 °C. At 7 days post-infection (dpi), infected cultures were either maintained in media favoring latency, or shifted into media favoring reactivation and co-cultured with naïve fibroblasts for an additional 14 d at either 32.5 °C or 39.5 °C. The frequency of infectious centers was then evaluated by an extreme limiting dilution assay (ELDA), the gold-standard for assessing and quantifying latent versus lytic/reactivated infections, as it quantifies the production of infectious progeny following latency [24]. *ts*C510G-infected CD34^+^ HPCs, both cultured and reactivated at the permissive temperature, reactivated to similar levels as parallel WT-infected cultures. Similarly, WT- and *ts*C510G-infected CD34^+^ HPCs, cultured at 39.5 °C for 7 d prior to shifting to 32.5 °C for the ELDA assay, revealed that these cells were capable of supporting viral reactivation at the permissive temperature. Importantly, while WT-infected HPCs produced an infectious virus following culture and reactivation at the non-permissive temperature (39.5 °C), the *ts*C510G failed to reactivate (Figure 3). In fact, the levels of infectious centers observed at the non-permissive temperature even prior to reactivation were lower than either WT or *ts*C510G at the permissive temperature (Figure 3), suggesting that maintenance at 39.5 °C reduced the background, spontaneous reactivation often observed in these assays. These data suggest that the IE2 C510G mutation (1) does not impact the ability of the virus to maintain latency or reactivate at the permissive temperature, and (2) impacts successful viral reactivation, similar to the impact on efficient lytic replication.

### 3.3. tsC510G-US28Δ-Infected Kasumi-3 Cells Have a Significantly Reduced Infectious Center Frequency When Cultured at the Non-Permissive Temperature

**Figure 4:**
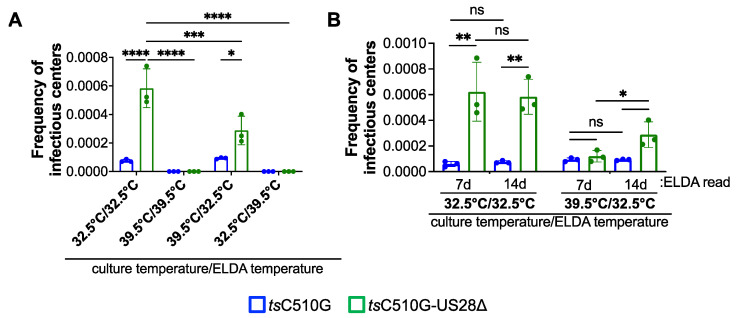
*ts*C510G-US28∆-infected Kasumi-3 cells display a decrease in the frequency of infectious centers at the non-permissive temperature. Kasumi-3 cells were infected (moi = 1.0 TCID_50_/cell) with *ts*C510G or *ts*C510G-US28∆ and maintained in conditions favoring latency at the indicated culture temperature (7 d). Each infected population was then co-cultured with naïve NuFF-1 cells (14 d) at the indicated temperature (ELDA temperature). (**A**) The frequency of infectious centers was calculated by ELDA 14 d post-co-culture. (**B**) The infected cells cultured at 32.5 °C and 39.5 °C that were shifted to 32.5 °C were quantified by ELDA at 7 d post-co-culture. Note that the 14 d data are replotted in this graph and identical to the data shown in (**A**). (**A**,**B**) Each data point (circles) is the mean of three technical replicates. Error bars indicate SD of three biological replicates. The statistical significance was calculated using two-way ANOVA followed by Tukey’s post hoc analysis. * *p* < 0.05, ** *p* < 0.01, *** *p* = 0.0005, **** *p* < 0.0001.

Since hematopoietic cells, such as Kasumi-3 cells and CD34^+^ HPCs, infected with viruses, in which US28 expression is abrogated or the US28 signaling domains are mutated, result in lytic rather than latent infection, there is a possibility that the cellular environment is significantly altered. We reasoned that a complete ORF deletion of US28 in the temperature-sensitive background may result in an infection with a reduced frequency of infectious centers at the non-permissive temperature. To determine if the IE2 C510G mutation could suppress the increase in infectious center frequency typically observed during US28Δ infection of hematopoietic cells, we infected Kasumi-3 cells with either tsC510G or tsC510G-US28Δ under latent conditions at either 32.5 °C or 39.5 °C for 7 d. We then co-cultured each infected cell population with naïve fibroblasts to evaluate the frequency of infectious centers by ELDA, and we maintained these cultures at the indicated temperatures for an additional 14 d. Consistent with previous findings, tsC510G-US28Δ-infected Kasumi-3 cells maintained at 32.5 °C for the entire assay displayed an increase in infectious center frequency compared to tsC510G-infected cells (Figure 4A), suggesting that tsC510G-US28Δ-infected cells failed to maintain a latent infection, in accordance with previous publications [10,11,12,15,16]. However, tsC510G-US28Δ-infected Kasumi-3 cells cultured at 39.5 °C prior to shifting to 32.5 °C for the ELDA displayed a significant reduction in the frequency of infectious centers (Figure 4A), suggesting the IE2 C510G mutation suppresses lytic replication in these cells. Regardless of the initial culture temperature, cells shifted to 39.5 °C for the ELDA yielded no detectable infectious centers, consistent with the data in Figure 3. While we did observe a statistically significant increase in infectious center frequency in the tsC510G-US28Δ-infected population compared to tsC510G cultured under the same conditions (Figure 4A), this is likely due to the fact these co-cultures were maintained at the permissive temperature for 14 d, thus allowing the tsC510G-US28Δ virus to replicate. We anticipated this, and thus we quantified the frequency of infectious centers by ELDA at 7 d, which revealed that the tsC510G- and tsC510G-US28Δ-infected cells resulted in similar infectious center frequencies (Figure 4B). Indeed, the tsC510G-US28Δ-infected cultures maintained at 32.5 °C for the duration of the entire assay already displayed a significant increase in the frequency of infectious centers by 7 d compared to tsC510G-infected cultures (Figure 4B), consistent with the fact US28-deletion viruses failed to maintain latency in hematopoietic cells. Together, these data suggest that maintaining tsC510G-US28Δ-infected Kasumi-3 cells at the non-permissive temperature forces the infection into a more latent-like state, thus preventing the production of infectious virus quantifiable by ELDA.

## 4. Discussion

Herein we generated two novel recombinant viruses, TB40/E*gfp*C510G (*ts*C510G) and TB40/E*gfp*C510G-US28Δ (*ts*C510G-US28Δ), which harbor the C510G mutation in the *UL122* sequence, shown previously to confer a temperature-sensitive mutation [22]. Our findings in lytically infected fibroblasts (Figure 1) confirmed previous findings from Heider et al., who showed this mutation in the CMV strain, Towne, resulting in the inability of the virus to replicate at the non-permissive temperature [22]. We also show that these newly generated viruses in the TB40/E background support the infection of hematopoietic cells, including primary cord blood-derived CD34^+^ HPCs and the CD34^+^ cell line, Kasumi-3 cells (Figure 3). Importantly, we demonstrate that *ts*C510G undergoes latency and reactivation at the permissive temperature, but not when maintained at the non-permissive temperature (Figure 3). Importantly, *ts*C510G-US28Δ fails to maintain latency under these conditions (Figure 4), consistent with previous findings [9,10,11,12,15,16]. However, we show *ts*C510G-US28Δ-infected Kasumi-3 cells cultured under latent conditions at the non-permissive temperature show a significant reduction in the frequency of infectious centers (Figure 4), suggesting that this virus could prove useful as a tool to study US28-specific regulated host and viral factors without inducing significant lytic replication.

CMV latent infection is maintained in HPCs and monocytes, while more differentiated hematopoietic cells, including macrophage and dendritic cells, support viral reactivation and lytic replication. The differentiation of both HPCs and monocytes down the lineage to the more mature cell types triggers viral reactivation. Thus, it is clear that the differentiation stage of the hematopoietic cell correlates with the infection stage of the virus. It is therefore possible that, due to the lytic-like phenotype that results from infection with a US28 mutant virus in HPCs and monocytes, the cellular milieu changes to one that supports reactivation and lytic infection, rather than latency. Such alterations in the cell environment could confound results, making it difficult to identify US28-specific changes versus those that may be due to a change in the cellular milieu.

We expect that the *ts*C510G parental backbone will be useful for other investigators who also study viral factors that are required for CMV latency. Indeed, this temperature-sensitive virus in hematopoietic cells may also be used as a tool for studying the events initiating viral reactivation and coincident cellular differentiation, as manipulating the culture temperature at defined times may provide temporal snapshots into these processes. We also anticipate this virus will prove beneficial to investigators interested in host–pathogen interactions at the initial stages of lytic infection.

## 5. Conclusions

In summary, we generated two new temperature-sensitive viruses to use as tools to study latency and reactivation, TB40/E*gfp*C510G (*ts*C510G) and TB40/E*gfp*C510G-US28Δ (*ts*C510G-US28Δ). We anticipate that the use of the *ts*C510G virus will prove beneficial to investigators interested in studying the impact of viral proteins that are required for latency.

## Figures and Tables

**Table 1:** Oligonucleotides used in this study.

Primer Use	Sequence (5′ to 3′)	Primer Name
Kan-I-SceI insertion (C510G mutation)	CGCTGCCACCCCCGTGGACCTGTTGGGCGCTCTCAACCTGGGCCTGCCCCTGATGCAAAAGTCGATTTATTCAACAAAGCCACG ^1^	IE2 C510G I-SceI 5′
	ACCATGACCTGTTTGGGAAACTTTTGCATCAGGGGCAGGCCCAGGTTGAGAGCGCCCAACACGCGTATATCTGGCCCGTACATCG ^1^	IE2 C510G I-SceI 3′
sequencing primers	GTGACACATCCACCCGAAGTGGCGCAGCGC	C510G US
	GTCTTCGGGAGGGGTCTCGGTGGGCTGCTC	C510G DS
*galK* insertion (US28Δ)	GGTGCGTGGACCAGACGGCGTCCATGCACCGAGGGCAGAACTGGTGCTATCCCTGTTGACAATTAATCATCGGCA ^2^	US28Δ galK 5′
	AGAGGGGCGGACACGGGGTTTGTATGAAAAGGCCGAGGTAGCGCTTTTTTATCAGCACTGTCCTGCTCCTT ^2^	US28Δ galK 3′
ds oligo	CAGACGGCGTCCATGCACCGAGGGCAGAACTGGTGCTATCTAAAAAAGCGCTACCTCGGCCTTTTCATACAAACCCCGTG	US28Δ ds oligo
sequencing primers	CCGCACATCTATTTTTGCTAATTGC	US28 fwr
	GCGACGAAACCCACCGTCACGG	US28 rev

^1^ Underlined sequences are complementary to the pEPKanS vector. ^2^ Underlined sequences are complementary to the *galK* template, pGalK.

## Data Availability

All data generated in this study are presented herein.
